# Review-Hysteresis in Carbon Nano-Structure Field Effect Transistor

**DOI:** 10.3390/mi13040509

**Published:** 2022-03-25

**Authors:** Yu-Xuan Lu, Chih-Ting Lin, Ming-Hsui Tsai, Kuan-Chou Lin

**Affiliations:** Graduate Institute of Electronics Engineering, National Taiwan University, Taipei 10617, Taiwan; r08943159@ntu.edu.tw (Y.-X.L.); d04943012@ntu.edu.tw (M.-H.T.); b99501013@gmail.com (K.-C.L.)

**Keywords:** nano-structure material, graphene, CNT, hysteresis, ambient condition, mechanism, factor, improvement

## Abstract

In recent decades, the research of nano-structure devices (e.g., carbon nanotube and graphene) has experienced rapid growth. These materials have supreme electronic, thermal, optical and mechanical properties and have received widespread concern in different fields. It is worth noting that gate hysteresis behavior of field effect transistors can always be found in ambient conditions, which may influence the transmission appearance. Many researchers have put forward various views on this question. Here, we summarize and discuss the mechanisms behind hysteresis, different influencing factors and improvement methods which help decrease or eliminate unevenness and asymmetry.

## 1. Introduction

Low-dimensional carbon (e.g., graphene and carbon nanotube (CNT)) displays extraordinary properties and shows great potential in many fields [1,2]. Graphene is two-dimensional material with sp2-bonded carbon atoms. As another allotrope of carbon, CNT is a one-dimensional material, and it could be envisioned as a rolled-up graphene sheet with diameters on a qa nanometer scale. Graphene and CNT display great mechanical, electrical and thermal properties, such as ultra-high elasticity, high electron mobility, tunable band gap and excellent thermal conductivity [3,4,5,6,7,8]. They are candidates for carbon nanostructure electronics and have accepted considerable interest from both academia and industry [3,5,9]. They can work as photodetectors, chemical sensors [10], biological sensors, etc. [11]. Apart from that, according to the 2012 International Technology Roadmap for Semiconductors, it is possible that CNT and graphene could replace silicon in technology and help extend Moore’s law after 2025 [2].

**Figure 1:**
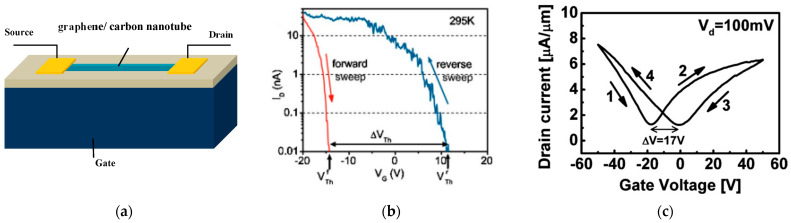
(**a**) Schematic structure of a back-gated carbon nanostructure FET [14]; (**b**) hysteresis characteristics in carbon nanotube FET at room temperature [9]; (**c**) hysteresis characteristics in graphene FET at room temperature [21].

What should be noticed is that device instability is one of the great challenges in the application of low-dimensional materials [12]. Hysteretic characteristics are a key issue of instability that we have to confront. CNT and graphene devices show gate hysteresis behavior in ambient conditions [13], which is not beneficial for the application of electronic transistors [14]. The usual structure of back-gated transistors with SiO_2_/doping-Si substrates is shown in Figure 1a. The typical hysteresis transmission of CNT and graphene is illustrated in Figure 1b,c. Commonly, the difference between the voltage of the lowest point (charge neutrality point) or the threshold voltage at the forward and backward curve is called hysteresis. The delaying width depends on many factors, including the device, the environment and measurements. Many researchers carried out a large number of studies, but no one has reviewed this phenomenon. Due to the fact that electronic applications require stable transport properties, it is important to summarize the mechanism behind hysteresis, factors correlated with hysteresis width and effective methods to fabricate hysteresis-free or hysteresis-control transistors in environmental conditions.

Similar levels of instability could be found in many other low-dimensional materials, such as graphene nanoribbon [15], MoS_2_ [16,17,18], WS_2_ [19], etc. More importantly, the mechanisms, factors and improvements referring to these nanometer materials resemble that of graphene and CNT. Researchers have found that hysteretic transportation of graphene nanoribbon (GNR) may relate to carrier trapping or detrapping processes at the interface of GNR and the substrate; this corresponds to graphene and CNT [15]. Similarly, hysteresis in MoS2 is associated with sweeping range, sweeping direction, sweeping rate, and thickness [16,17]. Meanwhile, they reach a consensus on encapsulation, which is helpful to fabricate hysteresis-free devices [18]. For example, with the encapsulation of 15 nm-thick Al_2_O_3_, hysteresis and threshold voltage shifts of MoS_2_ become smaller by 1–2 orders in magnitude [20]. Thus, our research may be used as a reference for other two-dimensional materials.

In this paper, we summarize the mechanisms, factors and improvements of typical low-dimensional nanometer material (CNT and graphene). Because CNT is a one-dimensional semiconductor and graphene is a two-dimensional semi-metal [3], they have subtle differences in hysteresis. We also make a comparison between them. Firstly, it is necessary to understand fundamental mechanisms. Mechanisms which have been proposed in previous papers are classified into three categories as surface traps, interface traps and dielectric traps. The differences between CNT and graphene originate from their band structure, contact area and dimension. Secondly, factors related to the hysteresis width are sorted into device characteristics (diameters and thickness, dielectric thickness, numbers of layer, etc.), environmental conditions (temperature and humidity) and measurement parameters (gate sweeping rate, range of gate volt, source volt and measurement methods). These are in accordance with the mechanisms we present. Thirdly, many researchers have explored various improvements to fabricate hysteresis-free or hysteresis-control devices. On the one hand, deposition on the substrate, encapsulation on top and changing dielectrics are popular methods proposed in studies. What is interesting is that not all materials are suitable for the passivation layer—some (such as NaPSS for CNT FET) may enlarge gate width. The representative experimented material used for protection, and their corresponding hysteresis has been summarized in Appendix B, Table A1 and Table A2. On the other hand, improving the process in physical and chemical methods under specific control is beneficial to optimization, where heating and annealing under vacuum are the simplest methods. In addition, new fabrication processes (such as dry transfer, semi-dry transfer, print, etc.) are put forward with the aim of manufacturing hysteresis-free and high-performance devices.

## 2. Mechanism

**Figure A1:**
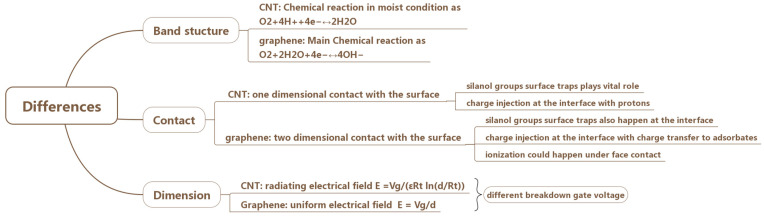
Different characteristics related to distinct mechanisms between CNT and graphene.

Many researchers proposed various mechanisms on the foundation of experiments and simulation work. According to the position of these mechanisms, we classify them into three categories, namely, surface traps, interface traps and dielectric taps. Evidently, surface traps take place on the surface of the device, interface traps occur between the material and substrate, and dielectric traps appear in the dielectric. Typical mechanisms under three categories of two materials are presented in Table 1. In this table, √ means the possible mechanism which has been proposed in the previous paper, and ○ represents the unlikely mechanism and has not been mentioned before. Overall, environmental components (such as different forms of water), residuals from the manufacturing process (residues from photoresist, organic solution, etc.) and initial defects all have an impact on hysteresis performance. We also summarize the different mechanisms between CNT and graphene due to their distinct characteristics in Appendix A, Figure A1.

### 2.1. Surface Traps

Surface traps is the most popular mechanism that proposed frequently in previous papers. It mainly comes from electron transmission in chemisorbed water, physisorbed water and silanol groups.

#### 2.1.1. Chemisorbed Water

Chemisorbed water molecular is the crucial cause of hysteresis [14,19,22,23,24,25,26,27,28,29,30,31,32]. The combination of O_2_ and H_2_O plays a key role in the doping process, and the electrochemical redox reaction is [31,33,34,35]:

O_2_ + 2H_2_O + 4e^−^ ↔ 4OH^−^ (1)

Several researchers simplified this equation [34,35] as H_2_O + e^−^ ↔ H_2_O^−^

The other kind of reaction also occurs in acid conditions, such as [36]:

O_2_ + 4H^+^ + 4e^−^ ↔2H_2_O (2)

**Figure 2:**
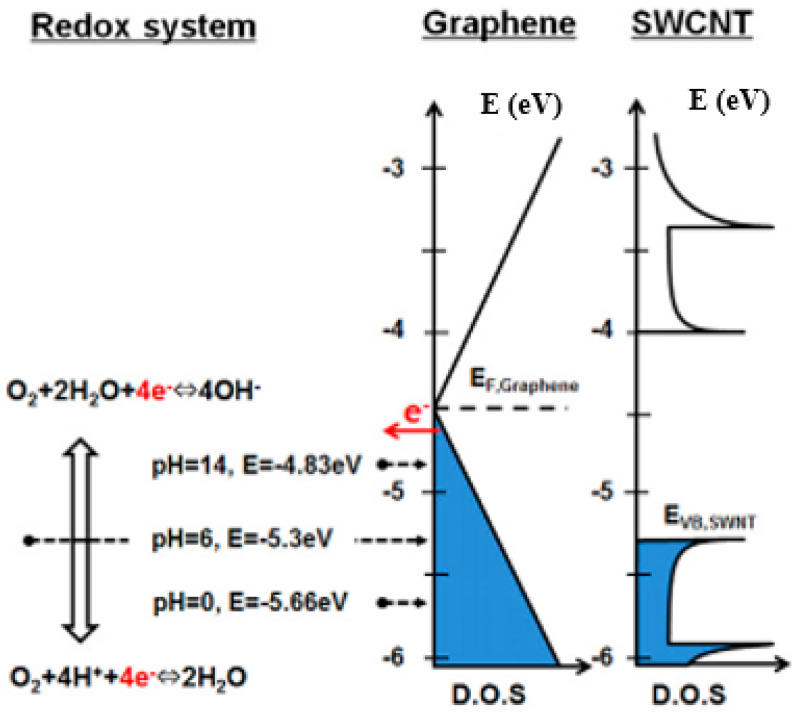
The density of state of the graphene and single-walled carbon nanotube with redox system [36].

The rate of reaction depends on the concentration of O_2_ and H_2_O; ΔG changes from −4.8 eV to −5.7 eV in alkaline (pH = 14) and acidic (pH = 1) conditions, as shown in Figure 2. Therefore, the reaction in Equation (1) was promoted when the density of OH^−^ was low [37].

The Fermi level of graphene lies at −4.5 eV, which is higher than the reaction potential in most pH values. The valence band position of small-diameter CNT lies roughly at −5.3 eV to −5.7 eV; this means that electrons transfer to the water layer in acid moist environments [11,38]. When applying the gate voltage to the device, the Fermi level shifts, and the charges are generated under different circumstances. For example, the Fermi level of graphene moves ±0.44 eV when applying ±40 V to the back gate device with 90 nm SiO_2_ [36].

Meanwhile, the electron-transfer mechanism is related to the Marcus–Gerischer theory [39]. The Fermi level and density of state (DoS) of graphene are changed with gate voltage, which can be calculated by the following equation as:

(3) $$n=\frac{{\varepsilon \varepsilon }_{0}}{{\mathrm{et}}_{\mathrm{ox}}}\left(V_{g}-V_{\mathrm{Dirac}}\right)=\overset{E_{F}}{\underset{E_{0}}{\int }}\left(2\left|E-E_{\mathrm{ig}}\right|/\pi \hbar^{2}\nu_{F}^{2}\right)\mathrm{dE}$$

where εε_0_ is gate dielectric permittivity, t_ox_ is gate thickness, V_g_ is gate voltage, E_ig_ means the intrinsic graphene Fermi level, E equal to the initial doping level and v_F_ is the Fermi velocity. V_Dirac_ is a constant and equal to volt at charge neutrality point. For intrinsic graphene, V_Dirac_ is 0 V. For doped graphene, it is related to residual charge n_0_ and can be calculated by V_Dirac_ = −n_0_et_ox_/εε_0_ at V_g_ = 0.

Therefore, the redox reaction at the surface and the uneven distribution of doping causes an inhomogeneous spread of the work function and influences the dynamic response of the graphene device under an applied back gate. This leads to hysteresis [30]. On the other hand, weak chemisorptions of O_2_ molecules also introduce possibilities of the doping in CNT and graphene [13,40,41].

#### 2.1.2. Physisorbed Water

Differently to chemisorbed water, physisorbed water presents layers of water droplets whose existence has been proved by environmental scanning electron microscope (ESEM) [42,43]. The amount of captured water changes flexibly with the relative humidity. They can bind electrons because of relatively high electron affinity (up to 0.8 eV) [43,44]. According to this, electrons on graphene would be trapped directly by the water layer and then diffuse to deeper droplets. These cause an electrical curve unbalance and hysteresis [43]. In CNT research, the existence of water molecules physisorbed onto the CNT surface is proposed simultaneously with chemisorbed water [14].

#### 2.1.3. Silanol Groups

Another significant hysteresis provider is silanol groups (≡SiOH) at the silicon oxide surface, especially for the supported device [26,45]. Silanol groups form with negative charges when the water molecules come into contact with SiO_2_. This phenomenon has been verified by using Fourier transform infrared spectroscopy (FTIR) [33,46,47].

**Figure 3:**
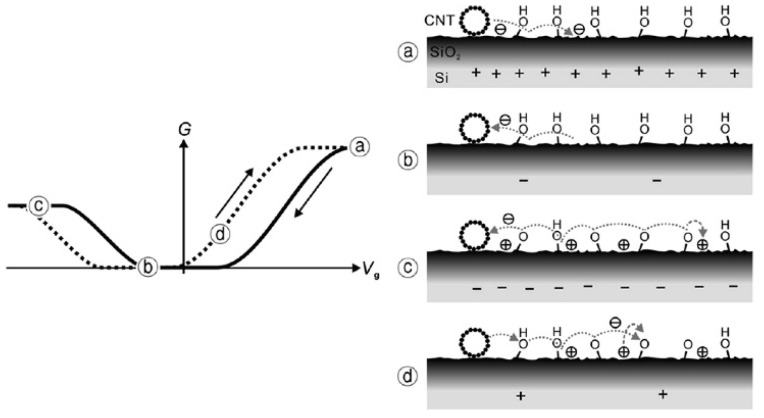
Diagram for silanol groups’ surface charging process [26].

In CNT and graphene research, the process of trapping and releasing protons is similar. As shown in Figure 3, silanol groups bonded on the silicon oxide are the charge traps. When the gate voltage is negative, silanol groups release protons and electrons. The lost protons may be trapped at nearby and the lost electron might be caught by nanotubes or by the electrodes. For the ionized silanol groups, the lost protons can also be transferred to water molecules as the proton absorbent [26]. This process is called field-driven hopping. The surface potential results of this process are kept up by scanning surface potential microscopy (SSPM). Another speculation also supports this model. The dielectric constant between H_2_O (ε = 80) and silicon oxide (ε = 3.9) is very different as the electrical field lines move from the plane capacitor to the water layer. The strong electrical field across the water layer results in the desorption and absorption of protons by terminal OH- groups [18,45,48]. Material initial defects lead to the rise in trap site density; this also promotes scatter and degrades mobility. The gate screening effect related to the existence of silanol groups on the surface is also a proposal. Because of the existence of ≡SiOH, charges accumulate on the SiO_2_, causing screening, hence resulting in hysteresis [19].

### 2.2. Interface Traps

**Figure 4:**
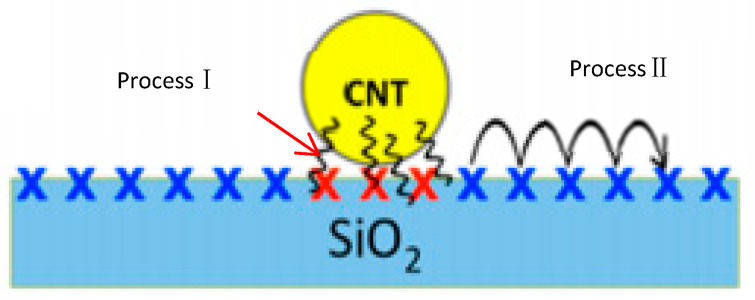
Transfer behavior with two steps of graphene [50].

It is commonly believed that the trap and release of surface charge take part in the process of charge transfer, which is similar to the conventional semiconductor device [49]. The second kind of mechanism for hysteresis is interface traps, which are relatively deeper than surface traps and mainly occur at the interface between materials and SiO_2_. The diverse positions of interface traps (process I) and surface traps (process II) are displayed in Figure 4. In this cross-section figure, the surface traps are represented by hopping via capture/emission, as proposed before, and interface traps are represented by tunneling.

In this part, hysteresis performance associates with charge injection (tunneling at the interface, charge transfer to nearby traps and adsorbates [12,51], screening with the foundation of charge traps [22]) [52] and the ionization of water at the interface [53,54].

#### 2.2.1. Charge Injection (Tunneling at the Interface)

**Figure 5:**
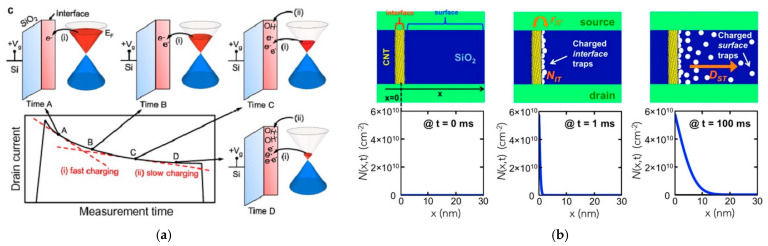
(**a**) Illustration of interface traps (tunneling) and surface traps (hopping via capture/emission) for graphene [12]; (**b**) illustration of interface traps (tunneling) and surface traps (hopping via capture/emission) for CNT [50].

In graphene research, many researchers believe that the decaying components of I_DS_ could be modeled as ΔI_DS_ exp(−t/τ) [55,56]. According to the model we proposed before, several researchers proposed the possible relationship between current and time with two different steps; the formula is shown below as Equation (4). It distinguishes the change of current into two processes, namely, fast charging and slow charging, as shown in Figure 5a.

(4) $$I=I_{0}\left[A\cdot \exp(\frac{-t}{\tau_{A}})+B\cdot \exp(\frac{-t}{\tau_{B}})\right]$$

where I_0_ is the initial drain current, A and B are parameters of process A and B, τ_A_ and τ_B_ the trapping time constant of two processes, respectively; t is the measurement time in this equation.

According to the fitting and calculation under vacuum or ambient conditions and with different temperatures, researchers find that process A is the dominant one, which occupied around 80% of the entire process and much faster than process B. In contrast, process B is very slow and has much lower influence in the process.

Based on above discussions, process A is related to the tunneling process, e.g., the transfer charge from graphene to nearby trap sites with weak activation energy and a short time constant. This process is dependent on the ambient temperature. On the other hand, process B is related to the interfacial redox reaction with high levels of activation energy, a large time constant [12,36,57].

This equation contains two time constants, τ_A_ and τ_B_ (τ_A_ ≈ 36.6 μs and τ_B_ ≈ 466 μs) [12]; these are much shorter than the trapping and detrapping time in the bulk SiO_2_ layer [58]; therefore, they are the representation of significant underestimation [21]. This formula represents the accumulation of electrons at the surface or the interface of SiO_2_ or the graphene channel [12,21,57].

For CNT hysteresis, similar tunneling is also proposed and calculated with the relationship between the density of charged traps and time and distance from CNT at the surface, which is related to different models as Si-OH surface traps and tunneling, as shown in Figure 5b [50]. At time = 0, no traps are charged. As gate voltage and time > 0, charges are injected. Then tunneling occurs and charges begin to be trapped at the interface. When the time at 100 ms, traps on the SiO_2_ surface diffuse and are charged within 10 nm away from the CNT. The obvious difference between two mechanisms in diffusion distance and velocity can be found in this mode [50].

What we should notice here is that tunneling for CNT has little difference compared with the tunneling proposed in graphene. Tunneling for CNT is almost independent of the temperature in ambient conditions. It increases a little with rises in temperature; this is equal to injecting charges with capturing protons. However, for graphene, tunneling has explicit dependence on temperature. Tunneling proposed in the graphene mechanism is similar to the charge transfer to nearby traps and adsorbates. Physisorbed water, silanol groups and adsorbates such as residues from organic solutions or photoreist- all work at the interface. The initial defect density also plays an important role [29,32,35,36,57,59,60,61,62]. In sum, tunneling sites possibly come from two candidates as graphene structural defects (such as combined dangling bonds) or adsorbates trapped at the surface of the graphene. These induce screening and scattering in graphene FETs [49,55,57,63].

Screening explains the charge injection from other aspects. Due to the change in injected electrons or holes at the CNT interface and reversal of the polarization charge in dielectrics under the mutative volt, the transistor shows the turn on or turn off state. The injected charges cannot dissipate immediately (the dissipation time is around 15 min) and the polarization induced by the gate bias changes rapidly; therefore, the dynamic screening effect results in hysteresis [9,22]. At a lower temperature, dipoles of water work on hysteresis. It can be oriented by electrical field and results in a change in carrier density by capacitive gating, which has keen competition with charge trapping [62].

Formulae may help us understand the process better. According to the Drude model, the equation of the drain current with rectangular graphene under low levels of V_DS_ is [59]

(5) $$I_{D}=\mu (\frac{C_{OX}C_{st}}{C_{OX}+C_{st}})(V_{G}-V_{Dirac})V_{DS}\frac{W}{L}$$

where C_ox_ and C_st_ are oxide capacitance and trap capacitance (capacitance caused by traps), respectively, L and W are the length and width of the graphene sheet, respectively, V_DS_ is the drain–source voltage, μ is the charge carrier mobility, V_g_ is gate voltage, V_Dirac_ is a constant and equal to volt at charge neutrality point.

Therefore, the extrinsic transconductance g_ex_ and intrinsic transconductance g_in_ (with no influence from traps) are shown in Equations (6) and (7) separately,

(6) $$g_{ex}=\mu (\frac{C_{OX}C_{st}}{C_{OX}+C_{st}})V_{DS}\frac{W}{L}$$

(7) $$g_{in}=\mu C_{OX}V_{DS}\frac{W}{L}$$

The ratio of the extrinsic transconductance g_ex_ and intrinsic transconductance g_in_ is

(8) $$\frac{g_{\mathrm{in}}}{g_{ex}}=1+\frac{C_{ox}}{C_{st}}$$

C_st_ is variable when measuring device characteristics. C_st_ is controlled by the trap density and gate volt, which determines the surface potential and also the possibility of occupation of the traps. The change in gate volt transform the graphene switches between two metastable conducting states [59].

#### 2.2.2. Water Ionization

Apart from the reaction, hysteresis has also been considered to be caused by ionization at the interface of graphene and the substrate [53]. Due to fact that the change of hole and electron density is affected by the different attached positions of hydroxide and hydronium ions on graphene and the substrate, free electron density restarted and hysteresis was formed [53]. Researchers also proved that polarized water has the tendency to absorb electrons from graphene, which corresponds with the fact that negative charges on oxygen shorten the O:H bond and extend the H–O bond [64]. In addition, the formation of C-O is proven to transfer charges from graphene to the substrate, which leads to the p-type of graphene [53,65,66].

### 2.3. Dielectric Traps

It is commonly believed that dielectrics have surface traps and bulk traps. Interface traps are what we have proposed before, and bulk traps related to dangling bonds exist in the oxide. According to calculations, the effective trap densities for the interface are N_it_ ≈ 5 × 10^10^ cm^−2^ and for the oxide are N_ot_ ≈ 5 × 10^11^ cm^−2^ [67]. Therefore, the interface traps at SiO_2_, namely, tunneling, and the oxide trap, namely, breakdown (avalanche or tunneling), exist simultaneously [9,50,68].

Because of the different shapes, graphene FET has a uniform electrical field and is calculated in Equation (9), but CNT FET has a radiating electrical field and can be calculated in Equation (10) [62].

(9) $$E=\frac{V_{g}}{d}$$

(10) $$E=\frac{V_{g}}{\varepsilon R_{t}\ln(\frac{d}{R_{t}})}$$

where d is the thickness of SiO_2_, ε the dielectric constant of SiO_2_ and R_t_ is the nanotube radius.

For graphene, oxide traps only occur when gate volt between 0.03 V·nm^−1^ to 0.27 V·nm^−1^ and SiO_2_ breaks down over 0.27 V·nm^−1^. For CNT, it can easily reach 1 V/nm, which is greatly larger than the breakdown field of SiO_2_ [62]. The high electrical field near CNT leads to pronounced hysteresis, even under a small range of voltage sweeping in the CNT transistor.

Since the avalanche needs a higher gate voltage than tunneling does, tunneling should also occur in graphene FET. However, few studies have explored tunneling in graphene. This might be due to the fact that previous studies typically use a smaller gate voltage than the breakdown voltage. Bulk charge trapping (~10^13^/cm^2^) in the region of good-quality SiO_2_ at low electric fields is unlikely to occur easily. Meanwhile, the measured time constant is too fast for the trapped center [45,58].

#### 2.3.1. Avalanche

**Figure 6:**
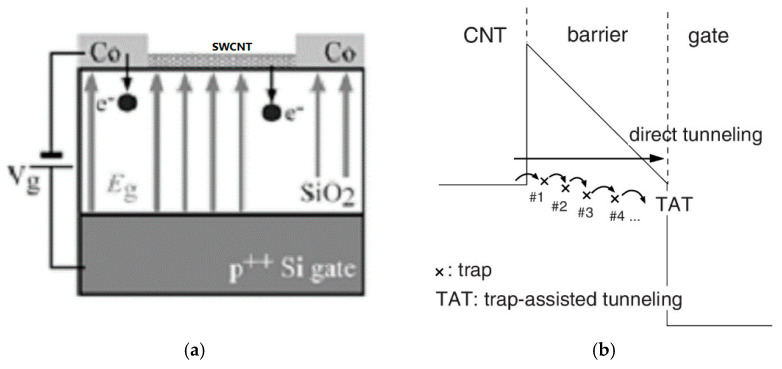
Schemes of dielectric traps: (**a**) Avalanche model [69]; (**b**) current injection model [70].

When the gate voltage is very high, avalanche electrons are injected from nanotubes into the bulk oxide and are kept trapped, as shown in Figure 6a. When the polarity and electrostatic environment are reversed, some of these electrons are released. Thus, both interface traps as a charge injection from the nanotube to the dielectric and surface traps we proposed before are co-responsible for hysteresis [69]. Similar avalanche injections are also mentioned in graphene devices [62].

#### 2.3.2. Tunneling and Trap Assisted Tunneling

Tunneling and trap-assisted tunneling (TAT) are proposed as supplements for bulk traps [69,70,71]. Direct tunneling is a mechanism that allows electrons to tunnel directly through the insulator of the barrier to the gate, whereas TAT means that carriers in barrier are captured and injected sequentially, as shown in Figure 6b. The influence of inelastic conduction (such as phonon emission) should also be considered to make the mode integral [70].

## 3. Factor

**Figure 7:**
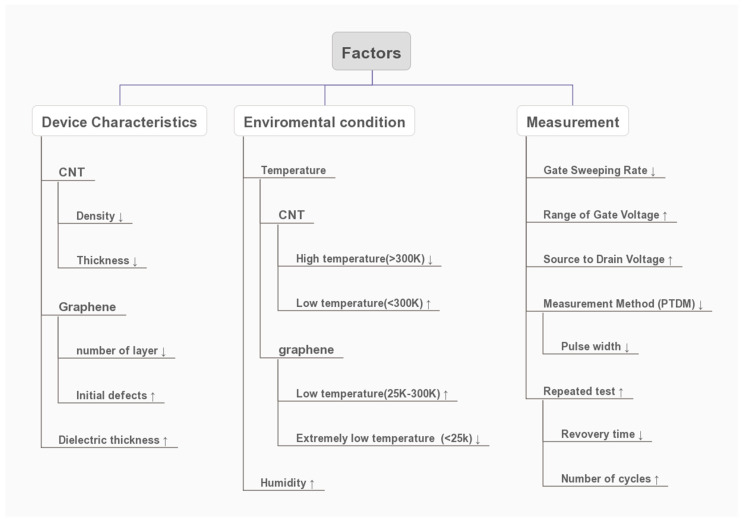
Simple Schematic of the Relationship between Factors and Hysteresis width (↑means positive correlation, ↓ means negative correlation).

Device characteristics (CNT density and thickness, graphene number of layers and dielectric thickness), environmental condition (temperature and humidity), measurement parameters (gate sweeping rate, range of gate volt, source volt and measurement methods) all have an effect on hysteresis width to varying degrees. A simple schematic of relationship between different factors and hysteresis width is shown in Figure 7. In this figure, ↑ means a positive correlation, and this, equal to hysteresis width, increases when the variable increase. Relatively, ↓ means negative correlation; this represents hysteresis drops with the variable decreases. The specific interaction is much more complex, which we will explain in detail below.

### 3.1. Device Characteristic

#### 3.1.1. Material Characteristics

**Figure 8:**
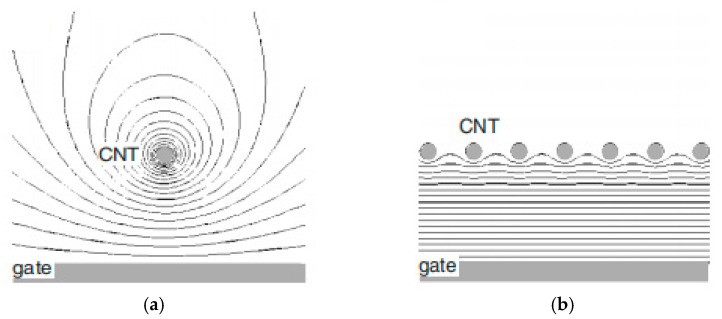
(**a**) Equipotential lines of isolated CNT; (**b**) equipotential lines of arrayed CNT [70].

For CNT, density and thickness are two factors which have been proposed, especially in simulation studies. The equipotential distribution of CNT is related to its density; this depends on whether it is isolated CNT or arrayed CNT. The line distance of isolated CNT is short around the CNT channel. For arrayed CNT, the line distance is equal and approximate to parallel [70]. Their equipotential lines are shown in Figure 8a,b, respectively. The line distance corresponds with CNT density. When CNT density is low, the distribution is similar to isolated CNT and its electric field can be estimated by Equation (10). The barrier height is associated with tunneling, which depends on Φ through Equation (11) [71].

(11) $$\Phi \approx \varphi_{CNT}-\chi_{SiO_{2}}-\frac{E_{G}}{2}$$

where ϕ_CNT_, χSiO_2_, E_G_ is the CNT work function, the SiO_2_ electron affinity and the CNT band gap at different diameter, respectively.

Meanwhile, when CNT density is high, the distribution is similar to CNT array and, much like a parallel capacitor, this does not match Equation (10). Apart from that, the electrostatic coupling and capacitance are influenced by distance due to the arrays of the CNTs [72].

The threshold voltage is influenced by the thickness of the CNT array, which means that the channel weakens the relaxation and increases the tunneling current. Threshold voltage has a positive correlation with CNT thickness. When the thickness is large, bottom CNTs which are near the gate and far from the top CNTs have opposite electric properties and screen top CNTs. This phenomenon leads to the relaxation of the electric force line and influence the result of hysteresis [70].

For graphene, the number of layers and initial defects are two main factors which should always be examined in studies. Researchers found that hysteresis has a relationship with the number of layers. The hysteresis decreases as the number of layers increase, which correlates with the charge distribution brought by interplay hopping and screening in multilayers [62].

Meanwhile, for graphene, the initial defect density and hysteresis show a linear growth relationship. It is an unignorable factor which can be calculated by Raman spectroscopy.

(12) $$n_{\mathrm{do}}=\frac{1.8\times 10^{22}}{\lambda^{4}}(\frac{I_{D}}{I_{G}})$$

λ_L_ is the excitation laser wavelength and the I_D_/I_G_ ratio originates from Raman spectroscopy [49,57]. Thus, dirac change drops as I_D_/I_G_ [57].

Surface trap density is dependent on the distance from graphene to the substrate and the position of the carbon atoms [13].

#### 3.1.2. Device Characteristics

In vacuum or under clean and dry conditions, we can estimate the trapped charges according to the dielectric trap mechanism. The relationship between trapped charges and the change in hysteresis can be expressed as [57]

(13) $$n_{t}=\frac{C_{OX}\times \Delta V_{Dirac}}{q}$$

where C_ox_ is the capacitance of the dielectric.

Dielectric thickness influences the capacitance of oxide dielectric. Thus, the trapped charges increase as the dielectric thickness decreases [49].

### 3.2. Environmental Condition

#### 3.2.1. Temperature

No matter what the mechanism is, they are all related to temperature. Capture probability is weakly related to $\sqrt{T}$ and emission probability is strongly related to $\sqrt{T}$ exp|(E_T_ − E_i_)/KT| for electrons and the hole [50]. Even though tunneling is independent of temperature, trap-assisted tunneling and electron distribution have a relationship with it.

**Figure 9:**
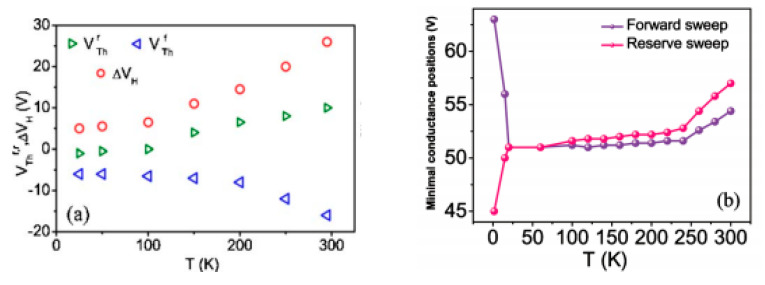
(**a**) Temperature-dependent hysteresis of CNT at lower temperatures [9]; (**b**) temperature-dependent hysteresis of graphene at lower temperatures [35].

Many experiments have been conducted to find the probable correlation between hysteresis width and temperature. For CNT, when the temperature is over 300 K, hysteresis width rises as the temperature drops [9,19,22]. What is unexpected is that they show a positive relationship under 300 K, as illustrated in Figure 9a (red dots represent the hysteresis value) [9]. This phenomenon may be due to the fact that the trapping/detrapping mechanism does not work for the mobile protons under low temperatures [26]. At that time, it just depends on the number of charges existing in CNT [22].

In graphene studies, researchers show more interest in hysteresis change under lower temperatures. Similar to CNT FET, there is a climb in the hysteresis for temperatures between 25 K and 300 K [35]. The difference is that the hysteresis loop of graphene FET changes direction under 25 K; CNT device behavior was not tested at this temperature range in previous studies.

As shown in Figure 9b, when the temperature is relatively high (almost over 25 K), the major loop is counterclockwise, which means that V_CNPF_ (charge neutrality point at the forward sweep)-V_CNPB_ (charge neutrality point at the backward sweep) is positive. When the temperature is high, the free electrons of graphene can be trapped by water at the interface. Therefore, water with trapped electrons turns into H_2_O^−^. When the temperature is low and further drops, these trap sites freeze. The hysteresis loop reverses to clockwise under low temperatures [35]. At that time, the reaction and trap states are not activated thermally, and this causes the reversion of ΔI_sd_. The electron-trap states turn into hole-trap states (frozen electron trap states), which can be expressed as h^+^+ H_2_O^−^ (ad) ↔ H_2_O. Hence, the number of electron-trap states at room temperature and hole-trap states at low temperature can be expressed by Equations (14) and (15) separately.

Mechanisms related to the electron-trap states net at room temperature [35,59]:

(14) $$n_{et}(T)=n_{Wt}\exp(-\frac{\Delta E_{t}}{kT})$$

At low temperatures, mechanisms related to the hole-trap states are as follows [35]:

(15) $$n_{ht}(T)=\eta n_{Wt}\left[1-\exp(-\frac{\Delta E_{t}}{kT})\right]$$

where T is temperature, n_wt_ means whole trap states density of dielectrics, ΔE_t_ is equal to the energy level of the trap states, k is the Boltzmann constant, η is the factor that describes the probability of the transformation from electron-trap states into hole-trap states.

Other researchers pointed out that the device shows obvious negative hysteresis at the low temperature of 0 °C, at which water may turn into ice at the surface. This may relate to their different sweeping rates in measurements. With a decreased sweeping rate, hysteresis increases and then becomes positive [62]. This experimental result connects with different dipole moments between ice and water layers.

Another interesting phenomenon corresponding with temperature is that the relationship between holding time at different temperatures and charge neutrality points displays various trends. When the temperature is below 400 K, the neutrality point decays with holding time due to the increase in the electron density. When the temperature is over 400 K, the devices show opposite behavior and display a slow increase with time. It is susspectd ionic species become mobile and thermally activated [55].

#### 3.2.2. Humidity

There is no doubt that hysteresis is dependent on humidity. The previous mechanisms proposed that hysteresis have correlation with water molecular. Humidity plays an important role in hysteresis, both in chemisorbed and physisorbed water. Hysteresis increases as the humidity rises and increasing speed decreases gradually as humidity value increases; even in the low vacuum condition, devices show similar characteristic [43]. Experimental results also prove that hysteresis increases significantly in vacuum, dry air and moist air [14]. For CNT, the fitted equation represents first-order exponential decay as V_th_ ∝e^−αh^, where h is related to humidity and α is constant [73]. The difference between the potential of CNT and graphene and their corresponding responses are shown before. For graphene, humidity relates to resistance change, which can be used as humidity sensors [74].

### 3.3. Measurement

#### 3.3.1. Gate Sweeping Rate

The width of hysteresis shows an obvious dependence with sweeping rate (equal to dVgs/dt) [23,49,75]. Hysteresis increases apparently as the sweeping rate decreases [45]. Even under a temperature of 0 °C, negative hysteresis also increases when the scanning rate goes up [62,76]. This phenomenon corresponds with the fact that the process of discharging at the surface takes longer than several seconds [14], and the trapping/detrapping process in the oxide also needs a process of reaction [62,75]. When the sweeping rate is slow, the carrier traps have enough time lag to finish the trapping/detrapping process, so in Equation (8), C_st_ is maximized, and the hysteresis result is minimal [59].

In addition, some researchers found that the forward curve is always higher than the backward curve in the I_d_–V_g_ figures. When the sweeping rate decreases, the forward curve drops but the backward curve remains almost constant. Eventually, two distinct branches overlap, and hysteresis diminishes [59].

#### 3.3.2. Range of Gate Voltage

The relationship between the gate sweeping range and hysteresis value is a curve with changed curvature, instead of a straight line [14,70,77,78]. Hysteresis increases with range of gate voltage and then saturates at certain voltages. This voltage corresponds to the density of available traps proposed in previous mechanisms [63,75,79]. Other researchers also found that the slope of line is bigger when the temperature increase; this is also consistent with the previous model. Their charge state changes when the gate voltage alters [77]. Additionally, on the basis of the surface charge exchange process, the loop direction depends on the polarity of the gate voltage (from negative to positive or from positive to negative) [35].

#### 3.3.3. Source to Drain Voltage

The dependence of V_DS_ on hysteresis is displayed on both materials [51]. Hysteresis width rises when V_DS_ increases due to surface traps [34,80,81,82].

#### 3.3.4. Measurement Method

The electrical measurement method and parameters have a crucial effect on the hysteresis result [63]. The pulsed time-domain measurement (PTDM) and repeated tests are two other common methods used in measuring, and they have an effect on hysteresis results.

PTDM is a method reliant on pulsed I_sd_–V_gs_ measurements [36,43,50]. Pulsed gate volt and width are two variables we should notice. Fermi levels are changed under pulsed gate volt and keep changing between the ‘off’ state and ‘on’ state [50,68]. Relaxation such as charging/emission occurs at intervals and gives the device time to return to its origin state; this process takes up to 0.1–10 s for CNT FET. Devices do not show free hysteresis characteristics until the off time is long enough to completely relax [71]. Electron mobility increases by 64% in this measurement method [21]. This measurement can also be used in other nanoscale devices.

Repeated test is another method where the device is tested repeatedly under different recovery times. In this method, recovery times and the number of cycles are significant. The time of the test has an apparent relationship with hysteresis [63]. In order to obtain a hysteresis-free device, we should allow traps to dissipate [22]. According to the experimental results, the residual interface electrons may stay in traps for over 500 s and keep accumulating at each loop, which enhances the effect of screening [63]. Because of this, Dirac point volt keeps increasing at the end of each repeated test. Due to the limitation of the trap sites, the difference of trapped charges shrinks at the interface and the voltage shift decreases as the cycles of back-and-forth increase [63]. Generally speaking, in the utilization of PTDM, we can control the hysteresis at different ranges or even obtain a hysteresis-free device by adjusting the time of relaxation and gate application. With different thicknesses of passivation, the control effect is more obvious. We illustrate it specifically in the following chapter. Relatively, repeated tests is the common method we used in experimental measurement; it is convenient and time-saving, but in actual fact this method causes charge accumulation and leads to larger hysteresis. Compared with PTDM, total recovery time is also comparatively larger if we want to eliminate the disturbance of hysteresis.

## 4. Improvement Way

In order to reduce the hysteretic behavior in the device, we should avoid contact with the following substances: (a) water molecules; (b) existing silanol groups on the surface; (c) residues which cause charge injects; (d) dielectric traps; (e) structural defects. Therefore, we summarize improvements into two categories, namely, composition change and process improvement. Composition change includes encapsulation on device, deposition on SiO_2_ and using an alternative dielectric layer.

The improving process contains thermal annealing, physical and chemical improvement and the new process.

### 4.1. Change Composition

#### 4.1.1. Passivation (Encapsulation)

For CNT or graphene FET, the method of encapsulation is of great help to solve electrical problems. As we discussed above, the water molecules remaining at surface is one of the reasons for hysteresis. Encapsulation on the top of device is an efficient way to tackle this problem. It also helps to improve device-to-device consistency and hysteresis variation [51,83].

##### Carbon Nanotube

There are many alternative materials working as candidates for encapsulation in this method for CNT. Several polymers, such as Teflon-AF (poly[4,5-difluoro-2,2- bis(trifluoromethyl)-1,3-dioxole-co-tetra-fluoroe-thylene]), CYTOP (Polyperfluoro- butenylvinylether), PMMA (poly(methylmethacrylate)) [14], Parylene-C [38,83], PVDF-TrFE (poly(vinylidene fluoridetrifluoroethylene)) [84], HMDS(hexamethyldisilazane) and OTS (octadecyltrichlorosilane) [51], have been discussed to act as the passivation layer in order to repel water at the surface. According to the result, Teflon-AF and CYTOP as hydrophobic fluoropolymers are effective in removing hysteresis completely [83]. This may originate from the fact that the dipolar nature of the fluoropolymer could neutralize impurities [84]. In particular, the device-passivated Teflon-AF with one hundred nanometers had stable hysteresis after 30 days of exposure to the environment or immersion in water for 24 h, which means that this device has excellent stability in dry air and even water. Apart from that, fluorocarbon passivation also has an impact on improving the uniformity of devices [83]. As a result of these fluoropolymer encapsulants, PMMA and parylene-C are unable to remove hysteresis fully [38,83]. The passivation layer of HMDS or OTS on both oxide and CNT shows a significant effect on the elimination of hysteresis [51] due to the removal of water molecules and the prevention of the formation of Si-OH [27]. The hysteresis value is still close to zero, even after re-exposing the coated device in a humid atmosphere for 24 h [51]. The encapsulation of PVP/pMSSQ (poly(vinylphenol)-poly(methyl silsesquioxane)) also proposed to change the threshold voltage, and that can also be used as a dielectric, which we discussed in the following page [85].

Material made in different ways exhibits different levels of sensitivity. In contrast to this kind of device, whose CNT was produced by drop-casting suspension, CNT fabricated by CVD shows more sensitivity to PMMA passivation in eliminating the gap and can obtain a hysteresis-free device within a certain gate range [14], which may relate to surface functionalization on the substrate, which causes a strong binding of water molecules [86]. However, experimental results prove that PMMA can provide ester groups to form hydrogen bonds with silanol groups. Moreover, the presence of PMMA as a hydrophobic layer prevents CNT itself from absorbing water [87]. However, hysteresis still exists with the larger gate volt and higher relative humidity that may be attributed to water and can permeate into the PMMA layer [12,78].

In addition, not all encapsulation helps to reduce hysteresis width. For example, the formation of NaPSS(poly(sodium 4-styrenesulfonate)) and Al_2_O_3_ could enlarge hysteresis; they can work as humidity sensors for a quick response within one second of a humidity change [88,89]. Using Teflon-AF as top gate which was used to modulate the threshold voltage for dual-gate operation [83] is another method which could be further explored in designing complex integrated circuits.

**Figure 10:**
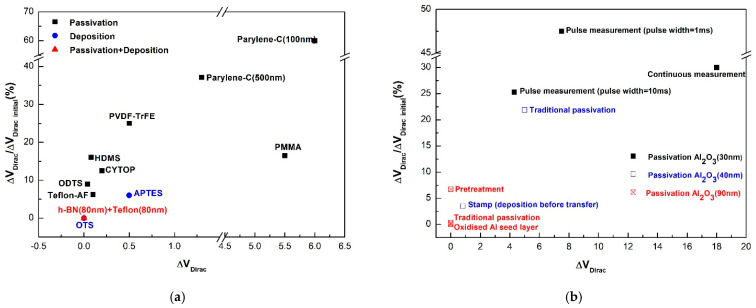
(**a**) CNT with different deposition and passivation materials [27,28,51,78,83,84,90,91]; (**b**) graphene with three deposition thickness of Al_2_O_3_ under different measurement and treatment [12,21,56,92,93,94].

Mainly, the encapsulation layer has two functions: draw water or remove electrostatic charge on the interface [85]. The comparison between ΔV_Dirac_ of typical deposition and passivation material we proposed in this paper is shown in Figure 10a. Choosing appropriate material for deposition and passivation helps to obtain hysteresis-free devices.

##### Graphene

In the case of graphene, researchers have tried various passivation materials, whereas many researchers concentrate on using Al_2_O_3_ as the encapsulation layer with a distinct process [59]. For graphene, ALD (atomic layer deposition) Al_2_O_3_ encapsulation [57] shows the opposite trend to CNT. It retards the chemical reaction process [12] and reduces the H_2_O molecules at the interface [36], which is attributed to helping eliminate hole doping and changing the dirac point [21]. According to the calculation result, larger passivation thickness could remove more hysteresis, as shown in Figure 10b [21,56,92,93]. Pulsed measurements with a larger pulse width also retard the hysteresis result [12,21]. This helps reduce the adsorbents and trapped charges produced in the fabrication process that deposits the ALD alumina layer with a thickness of 40 nm on graphene instead of deposition after transferring to the substrate [92]. Comparing the two results produced by these two processes, hysteresis could be eliminated and CNP reverts to zero from the positive voltage [92]. However, it is also possible that charges have interaction with deposited Al_2_O_3_ at the defect sites of graphene [21].

Other processes are proposed to eliminate the residues and block the charge trapping. Depositing the Al seed layer with oxygen chamber and then depositing Al_2_O_3_ on top is the process called oxidization of the Al seed layer [94]. In this way, Al might desorb molecules at the surface and compensate the original p-type doping of graphene [95,96]. Growing Al_2_O_3_ after pulses of pretreatment with O_3_ and H_2_O is the method that reduces hysteresis by achieving sufficient surface saturation on graphene and promoting nucleation [56]. Using deposited Al as the top gate is another way to fabricate zero-charge neutrality point FET [97]. Encapsulation not only improves the condition of hysteresis, but also increases the mobility value by 45–65% [36].

According to the experimental results, the deposition layer of Al_2_O_3_ has a negative effect on suspended CNT characteristics. Al_2_O_3_ deposition causes larger hysteresis for CNT [89], and this may connect with the H_2_O adsorption on the large hydrophilic surface of Al_2_O_3_ [98] or change of Pd contacts in the passivation process [89]. Apart from that, according to the comparison with studies on graphene, this may likely owe to the thickness of the Al_2_O_3_ deposition layer and the suspended structure of the device used in the experiment.

In addition, because of enhancement of the substrate dielectric constant and the neutralization brought from the amine group, PEI (poly(ethylene imine)) with increasing concentration in methanol solvent has an enhanced screening effect and hence has a neutral Dirac point and almost zero hysteresis [99]. While using the higher concentration of PET or other polarized dielectric, hysteresis direction may reverse because of capacitive coupling [62,99].

It is worthy to note that small residual hysteresis still remains after passivation. According to the classification of fast charging and slow charging, the contribution of the chemical reaction reduces significantly from 58% to 30% due to the process of encapsulation, but the charge exchange at the interface still exists, which is responsible for the remaining hysteresis [36]. Meanwhile, water molecules remain on Al_2_O_3_ in the formation of OH- groups and the water layer via H-bonding [98].

#### 4.1.2. Deposition Layer on SiO_2_

##### Carbon Nanotube

CNT-FET with passivation layer of OTS do not show gate hysteresis within certain measurement conditions. When giving a larger volt on gate, the hysteresis reappears in the opposite direction with a smaller value, which may be due to other mechanisms such as the residue charges in oxide being fixed, which could not be eliminated in this way [27]. Devices with a deposition layer of h-BN (hexagonal boron nitride) show similar characteristics that incompletely diminished hysteresis. This device with passivation can remove hysteresis more efficiently, which will be discussed below [51,90]. Modifying the substrate with APTES (amino-propyltriethoxysilane) before depositing CNT as self-assembled is another way to eliminate hysteresis. It can also be clearly found that CNT existed previously at the area, which has been modified. Additionally, in this way, hysteresis could be reduced immediately, becoming almost 15 times smaller than before deposition [91]. A small amount of hysteresis remains after heating the device to 80 °C in vacuum [91].

##### Graphene

Similarly, the deposition layer of HMDS functions as the hydrophobic layer and hysteretic behavior of graphene on HMDS primed SiO_2_ can strongly be suppressed [32], which is different to hydrophilic SiO_2_ [33,45]. HMDS as hydrophobic monolayers help reduce the formation of SiOH on substrate repress the adsorption process of dipolar molecules [32]. In comparison with empty substrate annealing at 300 °C, graphene on device with HMDS completely reverse p-type in few minutes more quickly than graphene on empty device. In this way, transfer charge from SiO_2_ to graphene still remains [59]. Deposition HMDS on SiO_2_ with anneal at high temperature in vacuum is effective to solve hysteresis problem after exposed to water in short time. However, in this way, scatter centers increase, hence carrier mobility reduces by 25% [45,100]. Compared with HMDS deposition result, self-assembled monolayer layer of OTS has smaller contact angle resulted in comparable smaller intrinsic doping [32].

Because of the substrate flatness and improved bonding at the interface [95,101], no charge transfer at the interface between graphene and h-BN, deposition layer of h-BN contributes to make device hysteresis-free and intrinsic [102]. Both the top and bottom h-BN gates eliminate p-doping and suppress hysteresis, which is also the result of no electrostatic charge transfer at the interface. Apart from that, graphene/h-BN FETs performs much-higher stability in comparison with graphene/SiO_2_ FETs [82,102]. However, it is difficult to achieve because large-area h-BN films are not yet available [103].

Other polymers are also used in deposition which is useful in solving hysteric problem. CYTOP (Polyperfluorobutenylvinylether) is a kind of amorphous fluoropolymer used to modify the SiO_2_ substrate with the aim of reducing the interface charge traps, and improving hysteresis and carrier mobility. Compared with unmodified devices, fluoropolymer modified devices show time-dependent stability and meet saturation at 4% in the first week instead of decreasing with time in ambient condition with relative humidity of 45% [104]. Parylene is helpful to improve characteristics [39]. Parylene is also considered as substitution of dielectric in graphene FET in order to eliminate ambient doping and decrease hysteresis caused by trapping [28]. However, according to the experimental results, it does not show better performance than previous films. In addition, deposited black phosphorus device shows free-hysteresis under the structure of SiO_2_ (bottom gate) and h-BN (top gate) [80].

Choosing appropriate film as deposition layer is the key point in order to improve transport. Another question we should consider in electrical model is that deposition way changes the equivalent oxide thickness (EOT) of bottom gate devices [51]. Therefore, the electrical field lines may deviate from plane capacitor [45].

#### 4.1.3. Both Passivation and Deposition

What the temporal evolution of devices is coating encapsulation layer on the top of device with deposition layer on bottom [90]. Compared with CNT FETs without Teflon encapsulation, device hysteresis with Teflon is much smaller [90], whereas it is interesting to note that hysteresis shows further obvious declination after several days to weeks, especially for 45 nm- and 80 nm-thick Teflon layers. Researchers also speculated that only when the thickness of Teflon is larger than 10 nm can this layer be effective to repel water [90].

It may also be more efficient to suppress hysteresis for graphene than using alternative substrates such as h-BN [105] or reoxidating SiO_2_ substrate, which helps solve this problem [106]; one such problem that we should consider is that graphene mobility is affected and limited by the substrate [106,107].

#### 4.1.4. Change Dielectric

##### Carbon Nanotube

Apart from deposition and passivation, changing dielectric contributes to alleviate this phenomenon. Polymer dielectrics (such as Teflon-AF, PMMA [78]) performs excellent electrical characteristics with a low level of dynamic charge traps and implies low hysteresis, which is similar to PVP/pMSSQ [85,108].

Dielectric layer called SiO_2_–Si_3_N_4_–SiO_2_ (ONO) layer which has high breakdown voltage, slow defect density and high charge retention capability [109] is also used to reduce hysteresis. In their design, tunneling can occur easily due to thinness of oxide layer between Si_3_N_4_ and CNT is small, the effective dielectric constant of this layer is almost 3 which means that it is easy to inject and extract charges [110]. Another one is HfO_2_−TiO_2_−HfO_2_ layer as dielectric instead of SiO_2_ which desorbs water slower than SiO_2_ [111]. In this way hysteresis width could remain stably to confirm the reliability [111]. Top gate (electrodes on top of CNT film) is another form in which devices with TiO_2_ (5 nm) as dielectric show hysteresis-free device under certain measurement [68].

##### Graphene

The water contact angles of SiO_2_, Al_2_O_3_ and Si_3_N_4_ are 14.3°, 27.9° and 42.9°, respectively. Considering the role water played in transmission, the Dirac point and hysteresis width of these dielectric devices have similar trends [82].

Top gate FET with the structure of h-BN/graphene/SiO_2_ and h-BN/graphene/BN shows high performance as suppressed hysteresis and p-doping [102]. Surrounding conditions with different dielectrics or solutions are also factors verified through experimental researchers and calculation [112,113,114].

Employing a high-κ substrate such as single-crystal epitaxial PZT(Pb(Zr_0.2_Ti_0.8_)O_3_) instead of SiO_2_ reduces Coulomb scattering with increased screening effects and improved transport characteristics, but no experiments have directly proven improvement in the hysteresis characteristic [112]. This kind of single-crystal epitaxial film could be discussed in future work.

### 4.2. Process Improvement

#### 4.2.1. Thermal Annealing

Heating [1,10,21], annealing [83] and vacuum pumping [14] play an essential role in the process which is attributed to the desorption of molecular adsorbates [51], especially for devices without encapsulation and deposition [115]. Bo Liu et al. made a comparison of the electrical performance of graphene after different thermal treatment, as shown in Table 2. The results remind us that we should also consider the thermal stability of different devices when choosing the temperature. For suspended graphene FET, thermal stability could reach up to 2300 °C in vacuum annealing [97,116]. However, thermal stability for supported graphene FET is much lower, at 100 °C, due to the strong interaction between graphene and the substrate. Graphene production method is one of the factors to notice. Chemical growth graphene has more initial defects than the physical exfoliated one. Therefore, it is more likely to be broken in annealing process. Generally speaking, choosing the appropriate temperature at 200 °C rather than higher or lower shows hysteresis-free behavior, owing to the removement or incorporation of solvent residues and amine surface functional groups [83]. Physisorbed water weakly absorbed on the material can be removed by pumping at room temperature for a period of time, but chemisorbed water hydroxylated with silanol groups can only remove SiO_2_ by vacuum annealing at temperatures over 200 °C [49]. In addition, annealing in different gas ambients such as Ar [41,86], vacuum [14,86], H_2_ [117], He_2_ [62], N_2_ [117], Air [114] and Ar/H_2_ [46] also leads to great results.

Annealing is effective in reducing hysteresis of CNT and graphene. For CNT, the characteristics of CNT FET turn into n-type behavior after heated in vacuum at 200 °C for about 20 h. It is suggested the n-type behavior is due to the interface charge transfer. This interface charget transfer helps to reduce the surface states [13]. For graphene, thermal annealing is significant in removing water molecules and making graphene intrinsic [36,102]. It is helpful to reduce hysteresis and remove the Dirac point, especially for multilayer graphene [41,43]. However, this method could not eliminate the trap in SiO_2_; because of this, hysteresis cannot be wiped out [62].

Annealing also plays a vital role for the device with passivation [51,83,118] Adding pre-annealing and post-annealing steps before and after the deposition of Al_2_O_3_ help optimize the process to achieving the symmetric transport and higher mobility and stability owning to removing molecules and absorbates at the interface or graphene channel [119,120].

Further researchers explored the most useful and suitable heating process. Hysteresis outcomes indicate that parameters such as temperature and time in the release and anneal process have a common effect on hysteresis decline. The releasing temperature contributes to removing resistance, and the annealing temperature contributes to desorbing water and absorbates on the surface [115].

Other methods such as rapid thermal annealing (RTA) and ultrahigh vacuum (UHV) could make the process more efficient. RTA with a temperature of 250 °C and a duration of 10 min set as the most effective parameters is helpful to make device hysteresis free [117]. Putting the device in a UHV whose pressure down to 10^−7^ Pa removes hysteresis entirely.

However, it is worth noting that although annealing in vacuum can greatly improve hysteresis width; exposing the device to atmosphere again introduces the possibility to rebound the hysteresis [82].

#### 4.2.2. Chemical and Physical Improvement

It is commonly believed that organic contaminations and residuals influence performance [104]. H_2_SO_4_ + H_2_O_2_ solution as the presentation of chemical improvement makes a contribution to the removal of residual impurities (such as photoresist) at the substrate surface [104,121], especially for the devices with a passivation layer, such as PMMA [121].

O_2_ Plasma is an effective physical method which proved to be able to change traps caused by photoresist particles on the electrodes and SiO_2_ substrate [57,68,122]. A longer process time results in smaller hysteresis [68], whereas according to the experiments, time > 20 min may damage the quality of CNT and inject defects. UV/ozone treatment and pentacene film deposition is another way to help diminish hysteresis width efficiently but incompletely. The remained hysteresis comes from the remaining purification or surfactant molecules, which has been proved by experiments with CNT FET [123].

For graphene, Ga ion irradiation has been certified as an effective way to degrade hysteresis by controlling the dose. When choosing the appropriate dwell time, most defects coalesced, resulting in a high tunneling barrier. Thus, the electron-trapping probability declined, and the hysteresis value degraded [54].

#### 4.2.3. New Process

Next, we introduce several new methods. For graphene, it is significant to avoid the contact with water and remove residues. Three categories as wet transfer, semi-dry transfer and dry transfer are proposed as main method in previous improvement. This is also appropriate to CNT FET. Apart from that, printing and self-alignment shadow mask are also the focus of attention. The comparison of hysteresis result by different methods are listed in the Appendix B Table A3 and Table A4.

##### Carbon Nanotube

The dry transfer method applies to CNT, which is similar to graphene. It optimizes the fabrication steps by laminating CNT and substrate by annealing and vacuumizing [78].

Printing as a new method is used to make hysteresis-free and flexible substrates by aerosol jet printing techniques or inverse gravure printing techniques, etc. [108,122]. Changyong Cao et al. developed the fabrication processes for completely printed CNT, in which ultra-thin polyimide film (Kapton) functions as the substrate and xdi-dcsis (a blend of PVP/pMSSQ) works as dielectric ink. Based on the water contact angle comparison, previous study demonstrated the contact angle of the spin-coated dielectric film (84.3°) is slightly smaller than that of the printed film (90.8°) [108]. It is suggested this contact-angle difference is because of the difference in surface roughness. Thus, this method is also helpful to eliminate the threshold voltage gap. Furthermore, extra advantages as excellent performance (such as high on-off ratio) and extreme bendability are available by this way [122].

The self-alignment shadow mask is a great candidate to deposit metal or the passivation layer. They have tapered contact geometry or suspended geometry so as to avoid charge traps or polar molecules caused by contact [89,124]. In addition, fabricating a metallic gate between the suspended area and dielectric with utilization of self-alignment is an effective way to avoid hysteretic phenomena. In this method, the influence of the oxide edge generated by the oxide substrate is diminished [23].

##### Graphene

**Figure 11:**
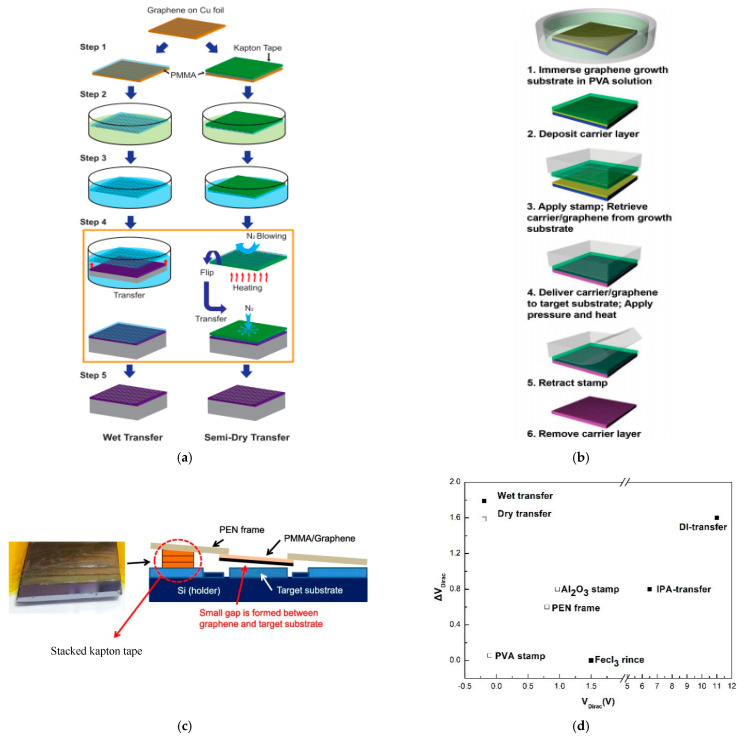
(**a**) Process of traditional wet transfer and semi-dry transfer [53]; (**b**) process of dry transfer [125]; (**c**) schematic of the structure to transfer graphene with PEN frame [95]; (**d**) ΔV_Dirac_ and V_Dirac_ of Graphene with new process of dry transfer and wet transfer [92,95,103,125].

We find that the process detail of separation and transferring is a key step in obtaining great performance devices. Therefore, we classify the treatments reported in previous papers into wet transfer, semi-dry transfer and dry transfer. The traditional transfer way is carried out by separating copper and graphene with the PMMA supporter by etchant solution or bubbles. Then, the film is transferred to the target substrate in water. Several researchers changed the transfer media from water to IPA and anneal in UHV at 300 °C; all these processes work as methods to remove the residual charges and change the interface bonding in order to improve transport performance, such as making the electrical curve symmetrical and reducing CNP difference [95,103]. Replacing deionized water with ammonia flow after etching copper film is proven to be an effective way to improve the electrical performance of the device. FETs display the zero Dirac point, symmetrical transport characteristics and better electrical mobility because of the existence of Fe^3+^ help remove the extra Cu^2+^ dopant [125]. Transferring graphene to the target substrate by using Kapton tape as supporter contributes to avoiding the attachment with water in the back-end process, which we called a semi-dry transfer [53]. The difference between the traditional wet transfer and the semi-dry transfer is illustrated in Figure 11a. This result demonstrates that the semi-dry transfer graphene FET has less p-type behavior and hysteresis [53].

Dry transfer and direct transfer avoid the contacts of graphene with water at the interface; researchers retrieved the graphene layer through carrier and stamp which are retracted after heating and pressure, as depicted in Figure 11b [92,95,125]. PVA (poly(vinyl alcohol)) and PMMA are two representative materials functioning as the supportive and protective layer. PDMS [125] or thermal release tape (TRT) work as stamp in FET fabrication. Similarly, Al_2_O_3_ deposition films is conductive to confirm the completeness and effectiveness of the transferred film [92]. The establishment of the PEN (polyethylene-naphthalate) structure (as shown in Figure 11c) is available for the transfer step under vacuum conditions [95]. The top frame is made by PEN and the supporting frame is made by kapton tap. The stacked kapton is used to control the gap between substrate and graphene. Under these conditions, voluntary bonding is initiated at a specific temperature range [95]. Apart from that, a thicker layer of ALD metal oxide (such as 100 nm of either alumina, hafnia or titania) can be used to transfer graphene directly in order to avoid the influence from polymer residues.

Finally, it is commonly believed that graphene grown through chemical methods, such as chemical vapor deposition, is more defective than mechanically exfoliated graphene, which causes more structural defects and results in hysteresis. Experiments have proven that it is possible to produce high-quality graphene, similar to exfoliated graphene, with careful control of the parameters in CVD growth with higher methane partial pressure [49]. Catalytic chemical vapor deposition (CCVD) is a novel method in which to directly grow bilayer graphene field-effect transistors, but the hysteresis width is large compared with other methods in the current study [61].

## 5. Conclusions

Gate hysteresis is a common phenomenon which can be observed in low-dimensional material (such as CNT, graphene, GNR, MoS_2_, WS_2_, etc.) that may not be beneficial to electronic transistors. In devices, hysteresis should be avoided or controlled. In traditional semiconductor device, hysteresis should be avoided or mitigated because of instabilities and non-uniformity introduced by hysteresis in integrated circuits. In contrast, controllable hysteretic behavior in 2D material FETs has great potentials for various applications, such as sensors and nonvolatile memory devices [16].

In this paper, we summarize several mechanisms related to the formation of hysteresis at surface, interface and dielectric, factors correlated with device characteristics, environment and measurement and also improvements against certain causes. The mechanism difference between CNT and graphene originating from these characteristics, such as band structure, contact and dimension, are illustrated.

For improvement, water molecules play a vital role at the surface and interface. Because of this, deposition and encapsulation could significantly remove hysteresis. It is worth noting that most of the deposition and encapsulation could not reach the aim of hysteresis-free devices, and sometimes water molecules can penetrate the cover layer, especially at higher temperature in ambient condition. In addition, because of the existence of the extra layer, the electrical field lines may deviate from the plane capacitor. This generates extra problems related to the model. Heating and annealing in a vacuum or in other gas ambient under controlled temperature is helpful to remove physisorbed and chemisorbed water molecules quickly and conveniently. However, it should be noted that the atmosphere gas molecules could also introduce hysteresis because of doping effects. It is clear that chemical and physical improvements reduce adsorbates such as photoresist. This kind of adsorbates could cause charge injection at the interface and it needs to be strictly controlled. A changing dielectric is one method to eliminate hysteresis induced by the oxide interface and bulk, which are related to tunneling at the interface and breakdown in the dielectric. Apart from that, many new processes such as dry transfer, semi-dry transfer etching copper, self-aligned fabrication method and jet print improve hysteresis to different degrees.

Overall, many researchers have explored distinct methods to remove hysteresis caused by the interface and the surface; almost no one try to control the delaying width by solving problems caused by three parts simultaneously. Fabricating hysteresis-free or hysteresis-controlled devices with high quality and a large area is still a difficult problem we need to solve and confront.

## Figures and Tables

**Table 1:** Summary of proposed mechanism for carbon nanotube and graphene in previous papers.

Category	Mechanism	Carbon Nanotube	Graphene
Surface traps	Chemisorbed water	√ * Chemical reaction in moist condition as O_2_ + 4H^+^ + 4e^−^↔2H_2_O	√ Main Chemical reaction: O_2_ + 2H_2_O + 4e^−^↔4OH^−^
	Physisorbed water	√	√
	Silanol groups	√ Mainly occur at the surface	√ Occurs at both surface and interface
Interface traps	Charge injection	√ Tunneling with protons	√ Tunneling with adsorbates
	Ionization	○ *	√ Attachment and detachment of ionized water at the interface
Dielectric traps	Avalanche	√ Occurs at relative low gate voltage	√ Occurs at relative high gate voltage
	Tunneling and Trap assisted tunneling	√	○

* √ means possible mechanism and has been proposed and ○ represents unlikely mechanism and has not been mentioned.

**Table 2:** Comparison of graphene in different thermal treatments [97].

Graphene/Substrate	Annealing Temperature	Annealing Gas Ambient	I_D_/I_G_	Dirac Point Shift
CVD graphene/SiO_2_	600 °C	Ar	0.32	0.15 V (top gate)
Exfoliated graphene/SiO_2_	400 °C	Ar	N/A	74 V (back gate)
CVD graphene suspended on TEM grid	~2300 °C	Vacuum	N/A	N/A
Exfoliated graphene/SiO_2_	300 °C	Vacuum	N/A	>80 V (back gate)
Exfoliated graphene/SiO_2_	400 °C	Vacuum	Negligible	>60 V (back gate)
Exfoliated graphene/SiO_2_	500 °C	Vacuum	Negligible	N/A
CVD graphene/SiO_2_	500 °C	Vacuum	~0.35	N/A
CVD graphene/SiO_2_	400 °C	N2	~0.3	>150 V (back gate)
CVD graphene/SiO_2_	560 °C	Air	~0.59 ± 0.10	N/A
Exfoliated graphene/SiO_2_	560 °C	Air	~0.61 ± 0.01	N/A
CVD graphene/SiO_2_	650 °C	Ar:H_2_ (9:1) at 133 mbar	~0.4	N/A

## Appendix B

**Table A1:** Improvement on hysteresis of CNT with deposition and passivation.

	Material	ΔV_Dirac_	ΔV_Dirac_/ΔV_Dirac initial_	Reference
Deposition	APTES	0.5	6%	[91]
	OTS	0	0%	[27]
Passivation	Parylene-C (100 nm)	6	60%	[38]
	Parylene-C (500 nm)	1.3	37.14%	[83]
	PMMA (spin coating)	5.5	16.50%	[78]
	PMMA (dry-transfer method)	1	3.00%	[78]
	PVDF-TrFE (≈100 nm)	0.5	25%	[84]
	CYTOP (90 nm)	0.2	12.50%	[83]
	Teflon-AF (100 nm)	0.1	6.25%	[83]
	Al_2_O_3_ (40 nm) (suspended under new process)	9	∝	[89]
	NaPSS Coating (<10 nm)	≈5	≈500%	[88]
Deposition and Passivation	h-BN (Bottom) (30 nm) + Teflon (Top) (40 nm)	1–3	38.1%	[90]
	h-BN (Bottom) (45 nm) + Teflon (Top) (40 nm)	<1	19.0%	[90]
	h-BN (Bottom) (80 nm) + Teflon (Top) (80 nm)	0.1	1.9%	[90]

**Table A2:** Improvement on hysteresis of graphene with deposition and passivation.

	Material	ΔV_Dirac_	ΔV_Dirac_/ΔV_Dirac initial_	Reference
Deposition	HMDS	≈0	≈0	[32]
	OTS	N/A	N/A	[32]
	Parylene-C (168 nm)	≈0	≈0	[39]
	h-BN + annealing	≈0	≈0	[102]
	CYTOP (7 nm)	3–4	17.5%	[104]
	black phosphorus	0	N/A	[80]
Passivation	Al_2_O_3_ (30 nm)	18	≈30%	[36]
	Al_2_O_3_ (30 nm) + Pulse measurement (pulse width = 10 ms)	4.3	25.3%	[21]
	Al_2_O_3_ (30 nm) + Pulse measurement (pulse width = 1 ms)	≈7.5	47.5%	[12]
	Al_2_O_3_ (40 nm) deposition after PMMA transfer	≈5	≈21.9%	[92]
	Al_2_O_3_ (40 nm) deposition before transfer (new process Al_2_O_3_ stamp)	≈0.8	≈3.5%	[92]

**Table A3:** Improvement on hysteresis of CNT with new process.

New Process	Specific Way	ΔV_Dirac_	V_Dirac_ (V)	Gate Range (V)	Reference
Dry transfer	PMMA transfer	1	≈24	−30–30	[78]
Full printed	Aerosol jet printing	≈0	≈5	−10–10	[122]
	Inverse gravure printing (bottom gate)	2.4	2.3	−40–40	[108]
	Inverse gravure printing (top gate)	0.23	−12.5	−40–40	[108]
Self-aligned shadow mask	Tapered contact pattern	0	≈20	−10–−25	[124]
	Contactless pattern	0	N/A	−20–20	[89]
	Self-aligned metallic gate	0	N/A	−2–2	[23]

**Table A4:** Improvement on hysteresis of graphene with new process.

New Process	Specific Way	ΔV_Dirac_	V_Dirac_ (V)	Gate Range (V)	Reference
Dry transfer	PVA stamp (PVA+PDMS)	0.057 (±0.026)	−0.11 (±0.17)	−3–3	[125]
	Al_2_O_3_ stamp (Al_2_O_3_ +PVA+TRT)	≈0.8	≈0.967	−40–70	[92]
	PEN frame (special stamp structure) in vacuum transfer	≈0.6	≈0.8	−40–40	[95]
Semi-dry transfer	Kaplon Tape stamp + (NH_4_)_2_S_2_O_8_ etching copper	≈20	19	−100–100	[53]
Wet transfer	DI-transfer	≈1.6	≈11	−40–40	[95]
	IPA-transfer+anneal in UHV at 300 °C	≈0.8	≈6.5	−40–40	[95,103]
	etching copper foil with Fecl_3_ rinse	≈0	1–2	−20–20	[125]
Growth improvement	CVD with higher methane pressure	4.81	25.64–20.83	−100–100	[49]
	CCVD	16.8 (±3.36)	−6–11	−15–15	[61]
